# Rat Experimental Model of Myocardial Ischemia/Reperfusion Injury: An Ethical Approach to Set up the Analgesic Management of Acute Post-Surgical Pain

**DOI:** 10.1371/journal.pone.0095913

**Published:** 2014-04-22

**Authors:** Maria Chiara Ciuffreda, Valerio Tolva, Renato Casana, Massimiliano Gnecchi, Emilio Vanoli, Carla Spazzolini, John Roughan, Laura Calvillo

**Affiliations:** 1 Department of Cardiothoracic and Vascular Sciences – Coronary Care Unit and Laboratory of Clinical and Experimental Cardiology, Fondazione IRCCS (IRCCS: Institute for Treatment and Research) Policlinico San Matteo, Pavia, Italy; 2 Laboratory of Experimental Cardiology for Cell and Molecular Therapy, Fondazione IRCCS Policlinico San Matteo, Pavia, Italy; 3 Surgical Department, IRCCS Istituto Auxologico Italiano, Milan, Italy; 4 Department of Molecular Medicine, Unit of Cardiology, University of Pavia, Pavia, Italy; 5 Department of Medicine, Cape Town University, Cape Town, South Africa; 6 Department of Cardiology, IRCCS Multimedica, Sesto San Giovanni, Milan, Italy; 7 Center for Cardiac Arrhythmias of Genetic Base, IRCCS Istituto Auxologico Italiano, Milan, Italy; 8 Institute of Neuroscience, Comparative Biology Centre, University of Newcastle, Newcastle upon Tyne, United Kingdom; 9 Laboratory of Cardiac Arrhythmias of Genetic Base, IRCCS Istituto Auxologico Italiano, Milan, Italy; University of Illinois at Chicago, United States of America

## Abstract

**Rationale:**

During the past 30 years, myocardial ischemia/reperfusion injury in rodents became one of the most commonly used model in cardiovascular research. Appropriate pain-prevention appears critical since it may influence the outcome and the results obtained with this model. However, there are no proper guidelines for pain management in rats undergoing thoracic surgery. Accordingly, we evaluated three analgesic regimens in cardiac ischemia/reperfusion injury. This study was strongly focused on 3R’s ethic principles, in particular the principle of Reduction.

**Methods:**

Rats undergoing surgery were treated with pre-surgical tramadol (45 mg/kg intra-peritoneal), or carprofen (5 mg/kg sub-cutaneous), or with pre-surgical administration of carprofen followed by 2 post-surgery tramadol injections (multi-modal group). We assessed behavioral signs of pain and made a subjective evaluation of stress and suffering one and two hours after surgery.

**Results:**

Multi-modal treatment significantly reduced the number of signs of pain compared to carprofen alone at both the first hour (61±42 *vs* 123±47; p<0.05) and the second hour (43±21 *vs* 74±24; p<0.05) post-surgery. Tramadol alone appeared as effective as multi-modal treatment during the first hour, but signs of pain significantly increased one hour later (from 66±72 to 151±86, p<0.05). Carprofen alone was more effective at the second hour post-surgery when signs of pain reduced to 74±24 from 113±40 in the first hour (p<0.05). Stress behaviors during the second hour were observed in only 20% of rats in the multimodal group compared to 75% and 86% in the carprofen and tramadol groups, respectively (p<0.05).

**Conclusions:**

Multi-modal treatment with carprofen and tramadol was more effective in preventing pain during the second hour after surgery compared with both tramadol or carprofen. Our results suggest that the combination of carprofen and tramadol represent the best therapy to prevent animal pain after myocardial ischemia/reperfusion. We obtained our results accordingly with the ethical principle of Reduction.

## Introduction

Experimental microsurgery on rodents has become a fundamental tool for translational research over the last 20 years. The quality of surgical procedures is a crucial factor especially when the level of technological sophistication, currently required by laboratory investigations, is taken into account. Molecular biology, proteomics, microarrays and biochemistry need a high standard of *in-vivo* methodologies to assure the highest quality of results. Moreover, ethics in animal care is of primary relevance as the surgical procedures in small rodents can result in a high risk of infections and inflammation leading to suffering, equally or probably more than in other experimental preparations. Small rodents do not vocalize nor communicate in the same way as primates or other common pet species, leading to unsubstantiated beliefs that they are comparatively more robust to infections or suffering. Recently, scientists have begun to observe rodent behaviour more closely, and this has led to a greater acceptance that they may experience suffering; perhaps just as intensely as in many other species. Methods developed to quantify pain and suffering in laboratory animals, including rodents, have already provided greater understanding of the need to refine analgesic and anaesthetic protocols in order to minimise suffering [1]. Rodent use has increased considerably in the last 30 years and they are now one of the most commonly used species in the cardiovascular research field [2].

The ability to apply in rats diagnostic techniques similar to those used in humans renders rat models of myocardial disease very useful for preclinical studies [3], [4]. Catheterisation, hemodynamic measurements, echocardiography, histological examinations and biochemical procedures are common tools in cardiac pathophysiology research. The need to conduct such research, not only in an ethically acceptable manner, but also with the greatest translational relevance is accepted as an essential aspect in the success of this work. Advances in pre- and post-surgical care of animals, including the use of anaesthetic and analgesic protocols and aseptic techniques are also considered to be likely factors in further improving the scientific reliability of results. However, despite this, not much has been done so far to develop clearly and appropriate guidelines for use of animals subjected to myocardial infarction. The rationale of this study was to verify if the methods developed to quantify surgical pain and distress during laparotomy [1] could be applied in experimental myocardial infarction and to verify if carprofen and tramadol, published as effective analgesic drugs in laboratory rodents, could provide pain relief after cardiac surgery. Thus our goal was to determine the best practice, in terms of rat’s care, in this particular model. We than compared different analgesic approaches in myocardial ischemia/reperfusion (I/R) injury: pre-surgical tramadol or carprofen or a combination of the two drugs (multi-modal group). Carprofen is a non-steroidal anti-inflammatory drug, which veterinarians prescribe as a supportive analgesic for various painful conditions, including surgery [1]. Tramadol is an opioid-like analgesic with a potent analgesic effects [5]. The goal was to establish which of the three approaches may reduce post-surgical pain and to apply the principle of Reduction to our experimental protocol.

## Materials and Methods

### Ethics

All procedures were approved by the Italian Institute of Health (Ministero della Sanità Italiano) (Permit Number 89/2009-B) according to 116/92 Italian Law and performed in accordance with the DIRECTIVE 2010/63/EU OF THE EUROPEAN PARLIAMENT AND OF THE COUNCIL on the protection of animals used for scientific purposes. The manuscript was prepared according to the ARRIVE (Animal Research: Reporting of In Vivo Experiments) guidelines [6].

### Refinement and Reduction

In compliance with the 3Rs principle of reduction, the work used rats already scheduled for studies on myocardial infarction, and the monitoring methods were designed to have no impact on the primary study outcomes. The present study focused on the first two hours after surgery as they were considered as the critical period when appropriate use of analgesia would have maximum positive impact on welfare.

### Materials

All surgical equipment was provided by 2Biological Instruments, VA, Italy. Drugs were provided by veterinary pharmacies: Contramal® (tramadol, Formenti, Italy), Rimadyl® (carprofen, Pfizer, Italy) and Amplital® (ampicillin, Pfizer, Italy).

### Husbandry

Male Sprague Dawley rats (n = 29) weighing between 250 and 295 g were supplied by Charles River (Calco, LC, Italy) and kept in cages with wood-shaving bedding (each cage (530 cm^2^) housed two rats). All cages were open to the room environment (no micro-isolation or ventilated caging) and rats were regularly handled by the staff involved in the experimental protocol. Room temperature was maintained at 21°±1°C with 50%±20% relative humidity and ventilated at 15 filtered air changes per hour. Animals were kept on a 12∶12 light:dark cycle and were provided *ad libitum* access to water and rodent feed. (Standard diet, Purina 5L79; Charles River, Calco, LC, Italy). Regular blood screenings on sentinel rats certified the absence of endoparasites and ectoparasites.

### Pre-surgical Preparation

Each researcher wore disposable clothes, gloves, footwear and a hair cover. An extractor fan was suspended above the surgical workspace as a barrier to extraneous airborne infectious particles.

The operating table, needle electrodes, heating pad and rat skin were disinfected with didecil-dimetilammonium chloride 0.175% (Farmasept, Nuova Farmec s.r.l., Italy) before surgery. The surgical tools were also sterilized with hot glass dry bead sterilizer.

Animals were placed on a rat/mouse rigid thermostatic pad and body temperature was continuously monitored using a rectal temperature probe (1 mm tip).

Ampicillin 100 mg/kg [7], was injected intra-muscularly, 2 minutes before surgery.

### Anaesthesia

Anaesthesia was induced with 4% isoflurane in 1.5 litres/min oxygen using a Perspex chamber and maintained with 2% isoflurane in 0.5 litres/min oxygen. We used isoflurane because this provides rapid induction and safe recovery from anaesthesia with relatively minimal effects on cardiovascular parameters or respiratory rate [4].

### Analgesia Experimental Protocol

**Figure 1:**
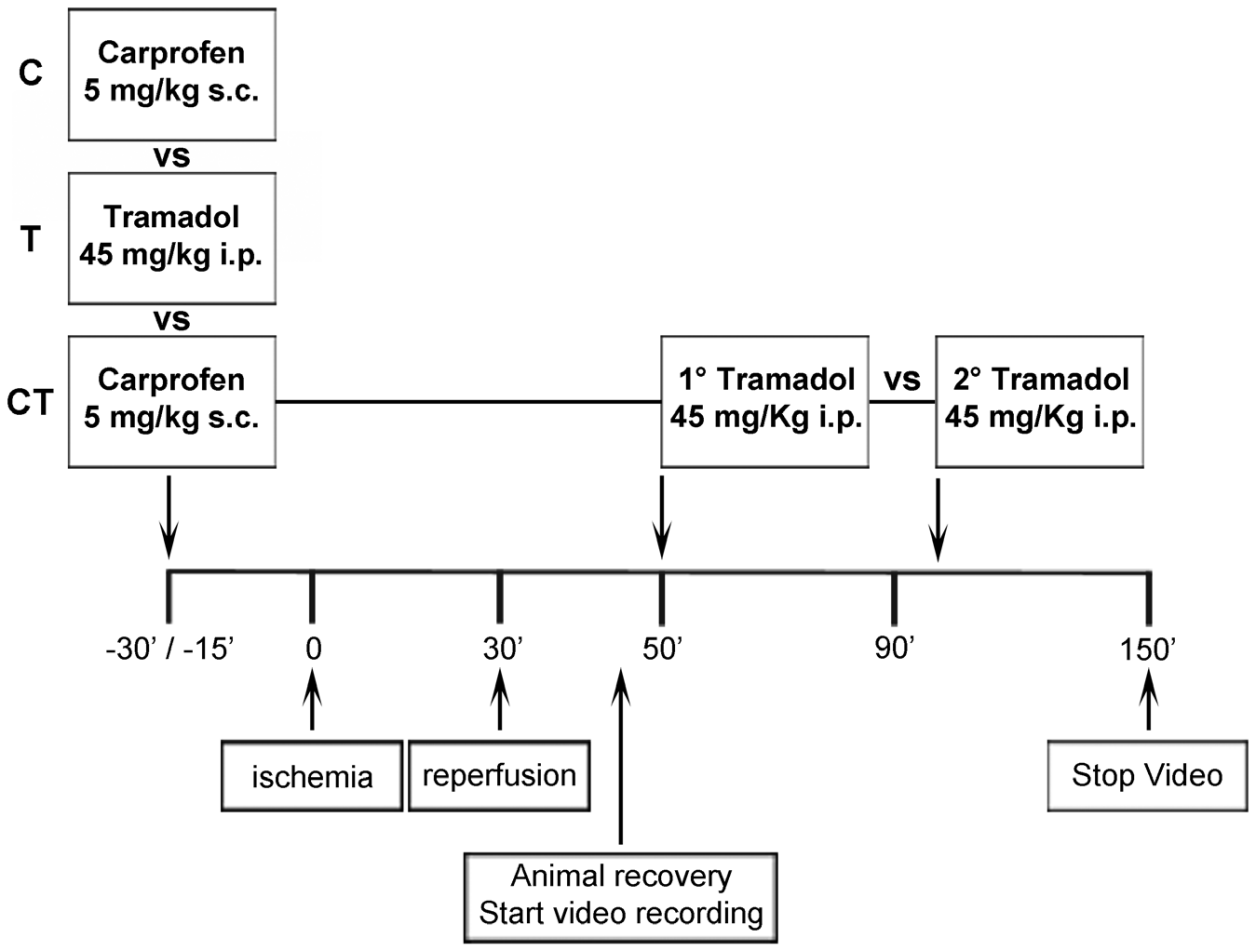
Experimental design. Three different analgesic treatments were compared: (C) 5 mg/kg carprofen (s.c. injection 30 min before surgery), (T) 45 mg/kg tramadol i.p. injection 15 min before surgery). (CT) Pre-treatment with 5 mg/kg of carprofen (30 min before surgery) and 2 i.p. injections of tramadol 45 mg/kg each (immediately after recovery of post-surgical mobility, then 1 hour later).

Three different analgesic groups were compared (**Table 1** **,** **Figure 1**):

**Table 1:** Non-standard Abbreviations.

C	Carprofen
T	Tramadol
CT	Carprofen+Tramadol
I/R	Ischemia/Reperfusion injury
s.c.	Sub-cutaneous
i.p.	Intra-peritoneal
LAD	Left coronary artery

Group 1 (C): pre-surgery carprofen, 5 mg/kg subcutaneously (s.c.), 30 min before surgery [1] (n = 9).
Group 2 (T): pre-surgery tramadol, 45 mg/kg intraperitoneally (i.p.) 15 min before surgery [8] (n = 9).
Group 3 (CT): combination of carprofen (30 min pre-surgery; 5 mg/kg s.c.) and two tramadol injection post-surgery (first injection (45 mg/kg i.p.) after recovery of mobility; second injection (45 mg/kg i.p.) 1 hour later) (n = 11).

The tramadol dose was selected on the basis of several published reports of its effective use in several invasive surgical models in rats [5], [9]. Considering that thoracic pain is one of the most acute among several surgical insults, we chose 45 mg/kg, the highest dose published [8].

All groups underwent I/R injury. For ethical reasons we did not consider conducting surgery in untreated animals or exposing rats to anesthesia unnecessarily, thus a saline control group was not used.

### Endotracheal Intubation

Under sedation rats were placed supine and a loop of 10 cm length of 3-0 silk-suture was placed around the upper incisors to fix the head. A fiber-optic light source was positioned about 1 cm above the anterior neck for the trans-illumination of the oropharingeal cavity.

The tongue was gently extended to bring the larynx into view. The intubation tube was made from an 18-gauge non-traumatic feeding needle connected to a Y-shaped connector attached to a rat ventilator. Respiration rate was 107 breaths/min; tidal volume 0.6 ml.

### Ischemia and Reperfusion

Five−lead ECGs (amplifiers Power Lab-ADInstrument Pty Ltd., UK) were recorded using 4 needle electrodes subcutaneously implanted in each hind/forelimb and 1 electrode placed ventrally on the right side of the chest. A tobacco-pouch suture was implanted through the pectoral muscles to allow rapid closure of the chest at the end of the procedure.

**Figure 2:**
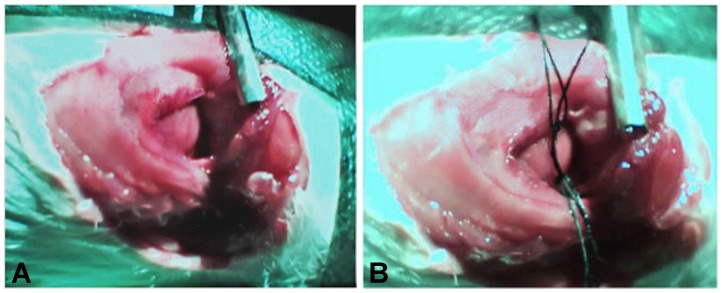
Left coronary artery ligation. (**A**) Heart visualization by the 15 mm opening of the 5^th^ intercostal space. (**B**) LAD was ligated with 4-0 silk suture and the plain knot was tied over two loops of suture. After 30′ ischemia loops were pulled and the knot was released allowing reperfusion.

The left coronary artery (LAD) was ligated using a 10 cm length of 4-0 silk suture material (Ethicon, Sommerville, NJ, USA) through a 15 mm opening at the 5^th^ intercostal space. A plain knot was tied and left in-situ for 30 minutes (**Figure 2**). Ischemia was confirmed in all rats by the appearance of discoloration of the heart surface and ST elevation on the ECG recording. Silk suture material was preferred to nylon as this prevented any slippage of the ligature. After 30 minutes the ligature was released [10] and reperfusion was verified by reddening of the previously discoloured area of the heart muscle and by the presence of arrhythmia on ECG recordings. The chest was then closed under negative pressure by gently squeeze of the ribs and pulling the pre-implanted tobacco-pouch suture. Rats were then extubated and observed for approximately 10 minutes under ECG and body temperature monitoring until they were able to move. Following this observational period they were transferred in cages for filming the post-operative behavior.

### Post-surgical Pain Assessment

#### Signs of pain

To assess behaviors indicating post-surgical pain, each rat was filmed for two hours in polycarbonate cages (810 cm^2^, Tecniplast S.P.A, Buguggiate, VA, Italy) using a webcam (QuickCam, Logitech). One observer, blind to the treatment, evaluated and counted in each rat predefined pain-specific indicators during the first and second hour after surgery; another researcher repeated the observations and counting for a double blinded check.

There were three main specific indicators previously shown to be effective in describing the presence of post-surgical pain and thought to reflect its severity [1], [11]:

1. “Stop” (later named as “Transient Stop”): scored in the absence of all other ongoing activity while rats adopted either a crouched or lying posture.
2. “Twitching”: transient involuntary muscular contraction of any body part usually occurring during ‘stop’.
3. “Stagger/fall”: loss of balance while walking and especially during rapid transition to crouch from high or low rear and/or during grooming.

We hypothesized that signs of pain would increase in response to surgery, but that their relative magnitude would reduce according to the various analgesic treatments tested. This would allow us to determine which treatment regimen afforded maximum pain relief.

#### Stress behaviors

In addition to pain-specific signs the observer made a subjective welfare assessment to verify whether there was evidence of general distress or suffering. The subjective score was based on whether there were commonly observed abnormal postures namely “Crouch hunch” and “crouch curl” [1], [11]. “Crouch hunch” was an abnormal posture during resting or ambulation with the rats head lowered and the back partially arched. “Crouch curl” was an exaggerated version of this with a crouched postured assumed in a hunched position such that the head was lowered between the forelimbs so that the eyes/snout were not visible.

In each case, the presence (yes) or absence (no) of suffering was assessed. A “yes” determination was considered when the animal showed these behavioral signs that the observer thought relevant, and only if lasted several minutes. A “no” evaluation was considered when the rats appeared active, normally responsive, calm, awake and resting normal without appearing agitated (despite the possible presence of signs of pain). The results were expressed as the percentage of rats assessed as ‘suffering’ in each group at each time point.

### Euthanasia

After recordings were completed the rats underwent additional manipulations as part of the investigations for the main study. Depending on that study requirements, rats were either euthanized by exposure to CO_2_ at a rate of 6 L/min in their home cages. We decided to use CO_2_ to eliminate the animal stress of handling. There is evidence that home-cage euthanasia is at least as acceptable as euthanasia in an induction chamber, as method of human approach, so whenever possible we chose this procedure [12]. In some cases rats were euthanized by induction of anesthesia with isoflurane (as in preparation for surgery) followed by heart explantation.

### Statistical Analysis

All analyses used SPSS Statistics version 19 (IBM). Continuous variables are presented as means ± standard deviation (SD). These were compared between groups using one-way ANOVA, followed by post-hoc multiple comparisons using the Tukey’s test or the Tamhane method whenever the assumption of homogeneity of variance (evaluated by the Levene test) was violated. Categorical variables were expressed as absolute and relative frequencies and analyzed using chi-squared tests. Within-group comparisons from the first to second assessment period (1 vs. 2 hours following surgery) were made using paired samples t-tests for continuous variables (2-sided). Alpha value was set at 0.05 for all analyses.

## Results

### Pain Monitoring

#### Signs of pain during the 1^st^ hour after surgery

**Figure 3:**
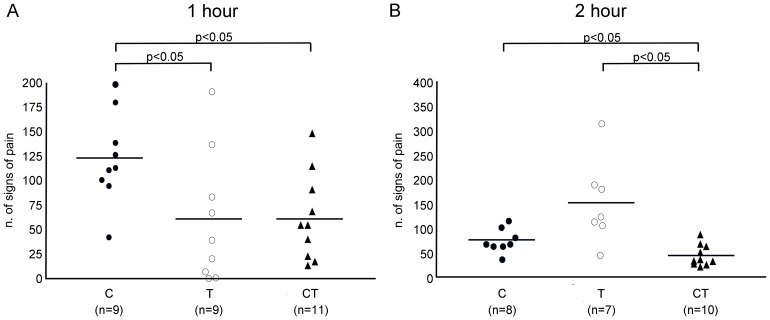
Pain assessment. Number of signs of pain during the first (**A**) and the second hour (**B**) after recovery from surgery. Horizontal lines represent mean values.

Our observations show that the ‘stop’ behaviour characteristically occurred during rearing and manifested as a transient ‘freezing’ of all ongoing activity. We hypothesize that this behavior is a transient reaction of few seconds in response to an acute ache. For this reason we named it as “Transient Stop”. “Twitching” occurred both during grooming and “Transient Stop”, and was the most common behavior that we observed. “Stagger/fall” and “Transient Stop” were less frequent compared to the other signs. ANOVA showed that during the first hour post-surgery the mean frequency of pain-specific behaviors varied significantly according to the treatment group (p<0.05); **Table 2**, **Figure 3A**). Compared to group C, where pain specific signs were most prominent (123±47), group T showed a significant reduction in pain-related behavior (60±67; p<0.05). However, rats treated with tramadol appeared narcotized for most of the assessment period. This effect was absent or considerably less pronounced when the first tramadol injection was administered following recovery (Group CT). The CT combination appeared to be more effective than C alone, with a significantly lower pain score (61±42 *vs* 123±47, p<0.05).

**Table 2:** Signs of pain and analgesic approaches.

Treatment	Time	Twitching	Stagger/Fall	Transient stop	total pains signs
Carprofen	1st hour	112±47	3±3	8±5	123±47*
Tramadol	1st hour	58±67	2±3	1±3	60±67
Carprofen+Tramadol	1st hour	59±42	1±1	2±2	61±42
Carprofen	2nd hour	70±23	0	4±3	74±24
Tramadol	2nd hour	129±87	0	22±33	151±86
Carprofen+Tramadol	2nd hour	42±20	0	1±1	43±21**

*p<0.05 *vs* T and CT (1^st^ hour);

**p<0.05 *vs* C and T (2^nd^ hour). Data are presented as mean ± SD.

#### Signs of pain during the 2^nd^ hour after surgery

Animals were completely recovered during the 2^nd^ observation period with the exception of 4 rats who died one hour after the onset of ischemia (one rat in group C, two in group T, and one in group CT). Signs of pain significantly increased in group T from 66±72 during the first hour to 151±86 at the second assessment time (p<0.05). Group C showed an opposite effect as in the second hour signs of pain significantly reduced compared to the first hour after surgery (from 113±40 to 74±24; p<0.05). In the CT group, the multi-modal treatment caused a small and not significant reduction in the frequency of pain-associated acts from the first and the second hour (65±42 *vs*. 43±21; p = n.s). However, the CT combination confirmed its superior analgesic effectiveness over a prolonged observation time as it resulted in an overall reduction of pain-associated behaviors compared to both the C (43±21 *vs* 74±24; p<0.05) and T (43±21 *vs* 151±86; p<0.05) groups (**Table 2** **,** **Figure 3B**).

#### Stress behaviors during the 1^st^ hour after surgery

**Figure 4:**
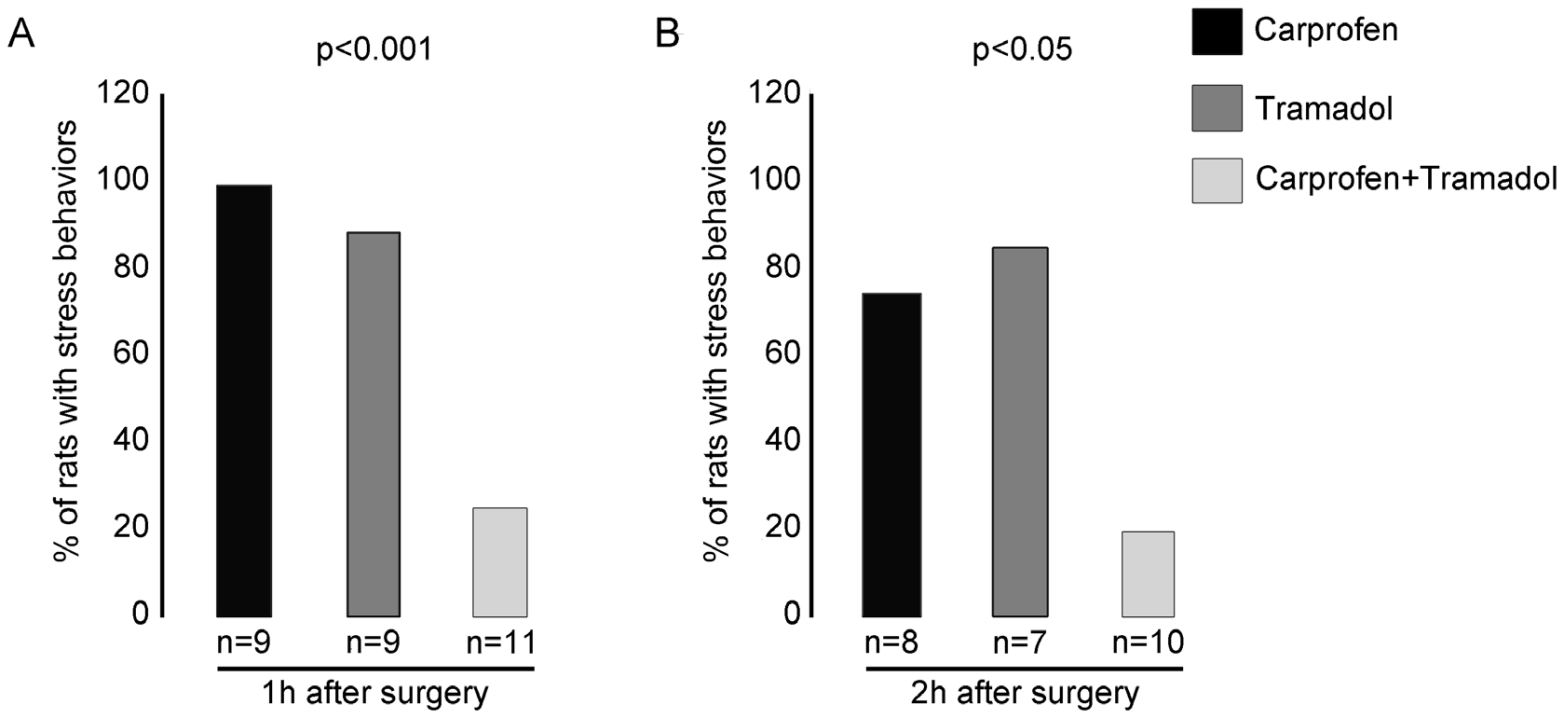
Suffering and distress monitoring. Proportion of rats showing stress behavior during the first (**A**) and the second hour (**B**) after recovery from surgery.

**Figure 5:**
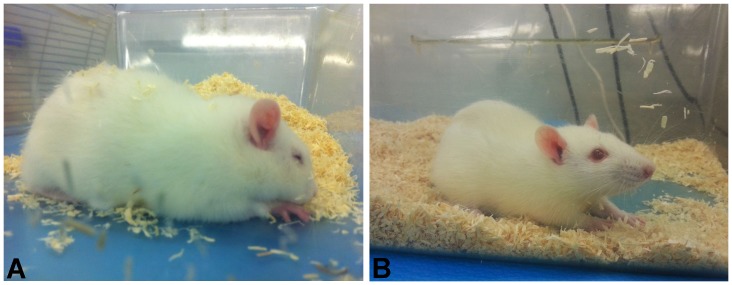
Example of stress behavior. (**A**) Carprofen treated rat exhibiting distress reactions 1 h after surgery. (**B**) Carprofen+Tramadol treated rat showing normal behavior 1 h after surgery.

As illustrated by **Figure 4A**, a significant (p<0.001) difference in the proportion of rats exhibiting the relevant signs was observed among the three analgesic groups. ‘Crouch hunch’ and ‘crouch curl’ were prominent in all rats in group C. In the T group, 8 out of 9 rats (89%) showed distress reactions but, as previously described, these animals were narcotized. It was therefore difficult to determine whether the reduced reactivity was a consequence of suffering or merely sedation. Again, the most effective treatment appeared to be the multi-modal combination of carprofen plus tramadol. Indeed, of the 11 CT treated rats only 3 (27%) showed abnormal behaviour (**Figure 5**).

#### Stress behaviors during the 2^nd^ hour after surgery

In the second assessment phase, the proportion of rats classed as suffering was relatively unchanged compared to the first hour of the study (**Figure 4B** **)**, and a significant (p<0.05) difference among groups was still observed according to treatment. Indeed, long periods of apparent distress remained relevant in the vast majority of rats in both groups C (6/8, 75%) and T (6/7, 86%). Conversely, only 2 of the 10 (20%) rats injected with the combined drug dose showed the relevant abnormal behavior.

## Discussion

The aim of the study was to assess a refined analgesic protocol for rats undergoing experimental thoracotomy and myocardial I/R injury. To accomplish our goal, we applied an effective method to assess pain and distress symptoms and evaluate the effects of different treatments providing pain relief to maximize post-surgical animal care. To test our protocols, we selected a highly traumatic procedure such as open chest I/R myocardial injury. We compared the effects of carprofen or tramadol given alone, with a combination of one dose of carprofen administered pre- and two doses of tramadol given post-operatively (multi-modal treatment). The primary methods of assessment were scoring pain-specific behaviors that accompany pain following other types of surgery (such as laparotomy [1]), and by using a more general (subjective) index of distress: posture and behavioral reactivity changes. Our results show that thoracic surgery caused effects consistent with post-surgical distress and generally poor welfare. Of the 3 groups tested, multi-modal treatment with carprofen and tramadol proved to be the most effective in reducing behavioral abnormalities over a prolonged observation. According to both methods of assessment, carprofen and tramadol given alone showed a relative lack of efficacy during the 2^nd^ hour following surgery.

### Pain Scoring

Although combined treatment seemed more effective, it was apparent that pain-specific behaviors and suffering were overall more pronounced than following laparotomy (unpublished observations). This justified our initial belief that surgery to study myocardial I/R may indeed be more painful to rats, as did the fact that none of the treatments completely prevented signs of pain. Also, back-arching, horizontal stretching and abdominal writhing behaviors, activities that can be used effectively to assess the severity of post-laparotomy and the efficacy of its analgesic treatment [1], were presently absent, whereas abnormal postures and twitching behavior were more frequent. This indicates that specific signs of pain and stress behaviors largely depend on the type of surgery undertaken. Thus, developing refinements to peri-operative care protocols that improve welfare requires the development of procedure-specific analgesic and pain scoring protocols and probably also refined husbandry practices (e.g. by careful handling and conducting precise surgery). Although our attempt to establish a pain-preventive protocol following myocardial I/R did not completely suppressed animal discomfort, to our knowledge this is the first study describing and evaluating the appropriate signs of pain and suggesting a refined treatment methodology for experimental thoracic surgery in the rat.

### Pharmacology and Efficacy

The lack of effectiveness of the pre-surgical treatment with carprofen or tramadol was probably affected by the route of administration and drug pharmacokinetics. Likewise in rats, Roughan and Flecknell concluded that 5 mg/kg carprofen administered subcutaneously had significant analgesic activity lasting between four and five hours following laparotomy [1]. Although, in our study carprofen had more pronounced activity at the 2^nd^ hour compared to the 1^st^ hour following surgery, it was still clearly inadequate as a standalone treatment.

The current observation of tramadol enhanced effectiveness, when given in the form of the combined therapy CT, shows this may be the drug of choice for more severe types of surgery. However, there are two problem issues regarding our use and assessment of the effects of tramadol; post-recovery apnoea and drug-induced narcosis. These effects of tramadol, especially the narcotic effects, may have affected our ability to effectively assess ‘suffering’. Those non-specific effects are commonly reported following opioid treatment in rodents, as recently reported in mice undergoing vasectomy following treatment with buprenorphine [13]. Accordingly, from our results it seems that when opioids are used, scoring proven pain-specific signs rather than evaluating generalized appearance provide a more reliable estimate of clinical efficacy. The problem was considerably less when tramadol was given following recovery (Group CT), presumably because the drug reached peak effect after the rat was completely recovered from anesthesia effects. The group treated pre-operatively with carprofen and post-operatively with two doses of tramadol did not appear narcotized, even at the 2^nd^ hour assessment time. This indicated that the timing of treatment could have a significant effect on post-surgical appearance.

The effect of opioids or opioid-like drugs such as tramadol on the immune system presents another possible problem. In a Sprague Dawley rat model of incisional pain, pre-treatment with 1 to 20 mg/kg i.p. of tramadol causes a decrease of IL-6 levels in a dose-dependent manner however, when used in combination with carprofen (CT) had no effect on IL-6 [14]. On the other hand, in Fisher 344 rats 20 to 40 mg/kg tramadol given before laparotomy preserved Natural Killer cells (NK) and IL-2 from the usual depression seen as a consequence of post-surgical stress [15]. Such strain-specific effects are therefore an important consideration in choosing the analgesic combination that is likely to be effective. As a consequence, improving scientific validity may require studies devoted to determining the precise manner of response to surgery for the most widely used rat models (for example Wistar Kyoto, Spontaneous Hypertensive Rats, Fisher, Wistar and Sprague Dawley). Better characterization of the response to analgesics in these is therefore also becoming recognized as essential to progress on refinement, and a basic premise to progress on the 3Rs.

The effect of a chosen analgesic regimen on infarct size is another potential source of variation. When given intravenously at 12.5 mg/kg in male Sprague Dawley rats before the onset of ischemia, tramadol has been shown to have a myocardial protective function against 6 hours of ischemia caused by permanent coronary occlusion, and reduces infarct size by inhibiting NF-kB activation [16]. This mechanism is of primary relevance to studies exploring strategies to modulate infarct expansion, or those concerned with investigating the role of the immune system in cardiac ischemia and failure. In our preliminary studies (data not shown) we verified that tramadol treatment in both groups T and CT did not interfere with infarct expansion. Indeed infarct size did not differ between groups C, T and CT and the calculated infarct size was similar to the necrotic size typically observed in experimental murine models [3], [10], [17].

## Conclusions

The principles of Refinement and Reduction were at the core of our work. This was achieved firstly by performing our experiments in rats already undergoing surgery. Secondly, we identified specific signs of pain and a multi-modal treatment able to reduce post-surgical suffering and improve animal welfare after thoracotomy. We assume that this was obtained by reducing their experience of pain with a development of a new more refined analgesic strategy, for use in this particular surgical model. In the future investigators should aim to further reduce animal suffering. This will require efforts to establish the exact mechanism underlying such multi-modal dosing, and use of the most effective behavioral (or other) methods of pain assessment. Our results indicate such testing should focus on pain-specific outcomes rather than generalized demeanor (due to a relative lack of drug associated non-specific effects).

## Study Limitations

The main focus of our study was the 3R’s ethic principles, in particular Reduction.

The European Directive *63/2010* recommends that “… the number of animals used in procedures could be reduced by performing procedures on animals more than once, where this does not detract from the scientific objective or result in poor animal welfare…”.

Accordingly to this recommendation, our study was planned to test a procedure (the three different analgesic treatments) on animals already undergoing an experimental protocol, thus reducing the number of rats involved and avoiding further suffering. In this context, our protocol guaranteed that animals used to evaluate the post-surgical analgesia were under the same experimental procedure just for the first 2 hours. We were aware that this ethical approach would have caused some limitations which would have prevented some important measurements like corticosterone levels, body weight and food intake and heart rate over the time, being all these procedures further objective parameters to show pain relief [13], [18]. Nevertheless, considering the spirit of this manuscript, we feel to have reached the important issue of giving useful observation on analgesia avoiding the use of rats specifically for this aim. Our purpose was to offer a possible alternative way to collect suitable informations without causing further stress to animals.
